# Supplementary material for: Activity, structure, and diversity of Type II proline-rich antimicrobial peptides from insects
Source: EMBO Rep. 2024 Oct 16;25(11):27. doi: 10.1038/s44319-024-00277-5 (PMC11549390; doi:10.1038/s44319-024-00277-5)
Supplement: Supplementary file 3 — Expanded View Figures [file 44319_2024_277_MOESM3_ESM.pdf]

Expanded View Figures

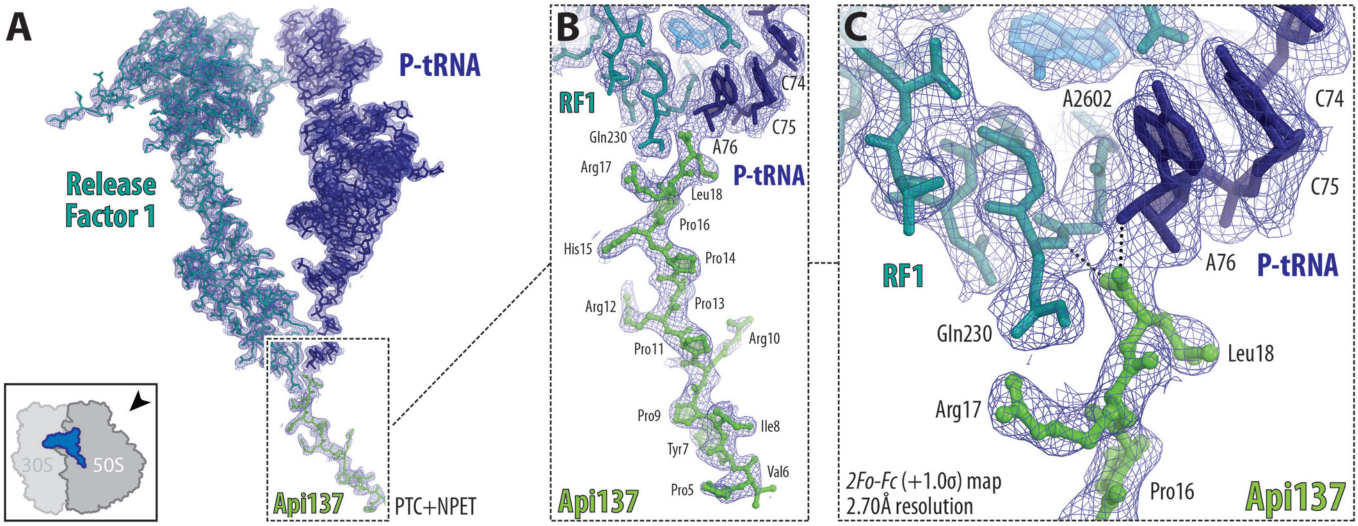

Figure EV1. Electron density map of the ribosome-bound Api137 peptide.

(**A–C**) 2F_o-F_c Fourier electron density map of Api137 in complex with the *T. thermophilus* 70S ribosome (blue mesh). The refined model of Api137 (green) is displayed in its respective electron density after the refinement contoured at 1.0σ. The adjacent ribosome-bound release factor 1 (RF1) and deacylated P-site tRNA (P-tRNA) are shown in teal and navy blue, respectively.

© The Author(s)

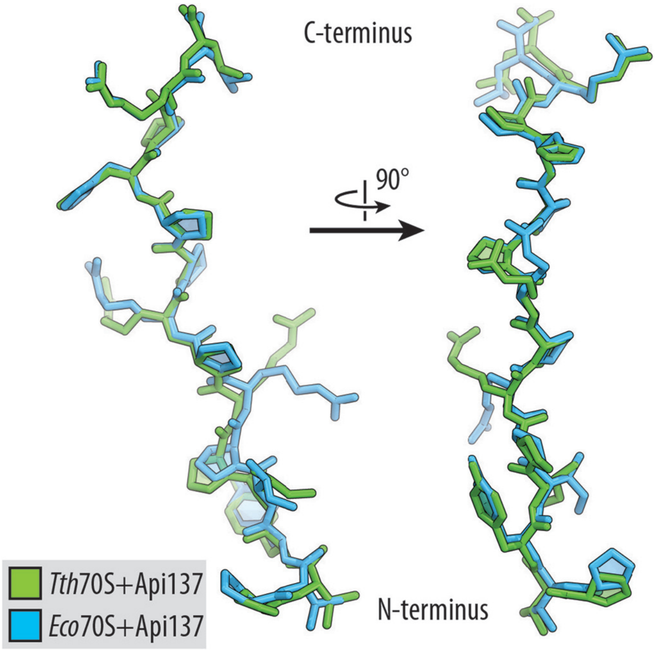

Figure EV2. Comparison of Api137 bound to *T. thermophilus* and *E. coli* 70S ribosomes.

Superposition of the new X-ray structure of Api137 in complex with the wild-type *T. thermophilus* ribosome (green) with the previous cryo-EM structure of Api137 in complex with ribosome from *E. coli* [blue, PDB entry 6YSS (Chan et al, 2020; Data ref: Chan et al, 2020)].

 © The Author(s)
